# Overcoming MDR by Associating Doxorubicin and pH-Sensitive PLGA Nanoparticles Containing a Novel Organoselenium Compound—An In Vitro Study

**DOI:** 10.3390/pharmaceutics14010080

**Published:** 2021-12-29

**Authors:** Letícia Bueno Macedo, Daniele Rubert Nogueira-Librelotto, Daniela Mathes, Josiele Melo de Vargas, Raquel Mello da Rosa, Oscar Endrigo Dorneles Rodrigues, Maria Pilar Vinardell, Montserrat Mitjans, Clarice Madalena Bueno Rolim

**Affiliations:** 1 Programa de Pós-Graduação em Ciências Farmacêuticas, Universidade Federal de Santa Maria, Av. Roraima 1000, Santa Maria 97105-900, RS, Brazil; leticiabuenomacedo@gmail.com (L.B.M.); danielamathes1609@gmail.com (D.M.); clarice.rolim@gmail.com (C.M.B.R.); 2 Departamento de Farmácia Industrial, Universidade Federal de Santa Maria, Av. Roraima 1000, Santa Maria 97105-900, RS, Brazil; josydivargas@gmail.com; 3 Departamento de Química, Universidade Federal de Santa Maria, Av. Roraima 1000, Santa Maria 97105-900, RS, Brazil; raquelmello.rosa@gmail.com (R.M.d.R.); rodriguesoed@gmail.com (O.E.D.R.); 4 Departament de Bioquimica i Fisiologia, Facultat de Farmacia i Ciències de l’Alimentaciò, Universitat de Barcelona, Av. Joan XXIII 27-31, 08028 Barcelona, Spain; mpvinardellmh@ub.edu; 5 Institute of Nanocience and Nanotechnology (IN2UB), Universitat de Barcelona, Av. Diagonal 465, 08028 Barcelona, Spain

**Keywords:** selenium compounds, pH-responsive nanoparticles, combination therapy, tumor cell lines, multidrug resistance (MDR)

## Abstract

In this study, we developed PLGA nanoparticles (NPs) as an effective carrier for 5′-Se-(phenyl)-3-(amino)-thymidine (ACAT-Se), an organoselenium compound, nucleoside analogue that showed promising antitumor activity in vitro. The PLGA NPs were prepared by the nanoprecipitation method and modified with a pH-responsive lysine-based surfactant (77KL). The ACAT-Se-PLGA-77KL-NPs presented nanometric size (around 120 nm), polydispersity index values < 0.20 and negative zeta potential values. The nanoencapsulation of ACAT-Se increased its antioxidant (DPPH and ABTS assays) and antitumor activity in MCF-7 tumor cells. Hemolysis study indicated that ACAT-Se-PLGA-77KL-NPs are hemocompatible and that 77KL provided a pH-sensitive membranolytic behavior to the NPs. The NPs did not induce cytotoxic effects on the nontumor cell line 3T3, suggesting its selectivity for the tumor cells. Moreover, the in vitro antiproliferative activity of NPs was evaluated in association with the antitumor drug doxorubicin. This combination result in synergistic effect in sensitive (MCF-7) and resistant (NCI/ADR-RES) tumor cells, being especially able to successfully sensitize the MDR cells. The obtained results suggested that the proposed ACAT-Se-loaded NPs are a promising delivery system for cancer therapy, especially associated with doxorubicin.

## 1. Introduction

Chemotherapy is the most common treatment for many types of cancer. However, the majority of the currently available chemotherapeutic agents show poor specificity by the tumor site, causing significant side effects and drug resistance [1,2,3]. In this regard, it is of the utmost importance the development of new compounds as well as new drug delivery systems to achieve a more effective antitumor treatment.

Nucleoside analogues represent an important class of chemotherapeutic agents used for the treatment of patients with cancer [4,5]. They are synthetic compounds, analogues of natural nucleosides, which act as antimetabolites. Inside the cells, they are phosphorylated and converted to their respective nucleotide analogues, which inhibit intracellular enzymes such as DNA polymerase or ribonucleotide reductase, as well as by being incorporated into newly synthesized DNA, causing inhibition of DNA synthesis. Nevertheless, resistance to nucleoside analogues are common and can occur by poor conversion into their active metabolites or by limited uptake by the tumor cells, due to a decreased expression of nucleoside transporter proteins [6,7,8]. Based on this background, new nucleosides derived from zidovudine containing chalcogenium are developed (5′-arylchalcogeno-3-aminothymidine, ACATs) [9]. Among these compounds, the 5′-Se-(phenyl)-3-(amino)-thymidine (ACAT-Se) present interesting antitumor potential; however, innovative pharmaceutical technological approaches are required to improve this activity.

The polymeric nanoparticles (NPs) present several advantages such as versatility, due to the possibility to customize the polymers, in addition to greater bioavailability, high encapsulation capacity for hydrophilic payloads, controlled release, good dispersion in water and ability to overcome the barrier of the lipid bilayer of the cell membrane by different endocytosis pathways [10,11,12,13]. Furthermore, after parenteral administration, the NPs can easily reach the tumor tissues because of the defective and leaky structure of tumor vessels, as well as the impaired lymphatic system. This phenomenon is known as enhanced permeability and retention effect (EPR) and allows the NPs to accumulate at the tumor site [14,15,16]. These characteristics make the NPs a promising approach to improve the efficacy of therapeutic cancer treatments [17]. Moreover, due to their elevated metabolism, tumor cells have slightly lower extracellular pH values (~6.5) than healthy tissues (pH 7.4), and this difference can be exploited to develop pH-sensitive drug delivery systems [18]. In this sense, our research group was studying a unique and exclusive group of anionic amino acid-based surfactants derived from N^α^,N^ε^-dioctanoyl lysine with pH-responsive behavior. They were incorporated into different NPs, assigning them a great potential to destabilize the endosomal membrane in mildly acidic environment [19,20,21,22,23].

In this study, to obtain an efficient drug delivery system for cancer therapy, we designed pH-responsive NPs incorporating 77KL (N^α^,N^ε^-dioctanoyl lysine with an inorganic lithium counterion) for the encapsulation of the organoselenium compound ACAT-Se. The NPs were prepared using poly(lactic-*co*-glycolic acid) (PLGA), a biocompatible, biodegradable and safely administrable polymer approved by the US FDA (Food and Drug Administration) and EMA (European Medicines Agency) [24,25]. In addition, poloxamer, a nonionic surfactant also approved by FDA [26], was used as a stabilizer and with the aim to increase the sensitization of tumors to the antineoplastic drug and to overcome multidrug resistance (MDR) in cancer cells [26,27]. The NPs were characterized and the role of pH in the membrane-lytic activity of NPs was evaluated using the erythrocyte as a model for the endosomal membrane. The in vitro drug release profile and scavenging properties of the NPs were also assessed. Furthermore, the safety of the NPs was evaluated by the hemocompatibility assay, and their nonspecific cytotoxicity was assessed using a nontumor cell line. The potential antitumor activity was assessed using sensitive and MDR tumor cell lines.

Finally, some studies suggest that selenium compounds present synergism with different cancer therapies, increasing the efficacy of the treatment and reducing the toxicity in the normal tissue [1,28,29]. In this context we evaluated if the NPs present synergic antitumor activity with doxorubicin (DOX), an antineoplastic drug commonly used for the treatment of a wide range of cancers [30].

## 2. Materials and Methods

### 2.1. Materials

Poly(d,l-lactic-*co*-glycolic acid) (PLGA, 50:50, 24–38 kDa), sorbitan monooleate (Span 80^®^), poloxamer 407 (Pluronic^®^ F-127), 2-2-diphenyl-1-picrylhydrazyl (DPPH), radical, 2,2′-azinobis-(3-ethylbenzothiazoline-6-sulfonic acid) (ABTS), 2,5-diphenyl-3-(4,5-dimethyl-2-thiazolyl) tetrazolium bromide (MTT), neutral red (NR) dye, phosphate-buffered saline (PBS), and trypsin-EDTA solution (0.5 g porcine trypsin and 0.2 g EDTA·4Na per liter of Hank’s Balanced Salt Solution) were obtained from Sigma–Aldrich (São Paulo, SP, Brazil). Fetal bovine serum (FBS) and Dulbecco’s Modified Eagle’s Medium (DMEM), supplemented with L-glutamine (584 mg/L) and antibiotic/antimycotic (50 mg/mL gentamicin sulfate and 2 mg/L amphotericin B), were purchased from Vitrocell (Campinas, SP, Brazil). All other solvents and reagents were of analytical grade.

5′-Se-(phenyl)-3-(amino)-thymidine (ACAT-Se) was obtained from the LabSelen-NanoBio (Federal University of Santa Maria, Brazil). This compound was synthesized and fully characterized as previously described (da Rosa et al., 2017).

### 2.2. Preparation of Nanoparticles

The NP suspension were prepared by a nanoprecipitation method [31]. Firstly, ACAT-Se (0.030 g) previously dissolved in methanol (6 mL) was added in a solution containing PLGA (0.050 g) and Span^®^ 80 (0.080 g) in acetone (30 mL), and this organic solution was kept for 20 min under magnetic stirring. Then, the organic solution was quickly poured into an aqueous solution (30 mL) containing Pluronic^®^ F-127 (0.150 g) and the pH-sensitive adjuvant 77KL (0.005 g). The pH of this aqueous solution was previously adjusted to 8.0 with NaOH 0.1 M (early pH~7.0). After 10 min under magnetic stirring (530 rpm), the organic solvent was eliminated by evaporation under reduced pressure to achieve 10 mL of final volume (ACAT-Se-PLGA-77KL-NPs).

NPs without the active compound (PLGA-77KL-NPs) and the pH-sensitive adjuvant 77KL (ACAT-Se-PLGA-NPs and PLGA-NPs) was also prepared for comparison proposes. All formulations were made in triplicate.

### 2.3. Characterization of Nanoparticles

The NP suspensions were diluted in ultrapure water (1:500 *v*/*v*) for the determination of the mean hydrodynamic diameter and the polydispersity index (PDI) by dynamic light scattering (DLS) using a Malvern Zetasizer ZS (Malvern Instruments, Malvern, UK). Each measurement was performed using at least three sets of ten runs. The zeta potential (ZP) was evaluated by electrophoretic mobility, using the same equipment. For this measurement, samples were diluted in 10 mM NaCl aqueous solution (1:500 *v*/*v*). Analysis of pH was done directly in the NP suspensions, at room temperature, using a calibrated potentiometer (UB-10; Denver Instrument, Bohemia, NY, USA).

### 2.4. Analytical Method

A reversed-phase liquid chromatography (RP-LC) method was developed for the quantification of ACAT-Se in the NPs. The method was performed, at room temperature, on a Shimadzu LC system (Shimadzu, Kyoto, Japan) equipped with an SPD-M20A photodiode array (PDA) detector, using a Gemini NX C18 Phenomenex column (150 mm × 4.6 mm; 5 μm). The UV detection was set at 263 nm and the LC system was operated isocratically, using a mobile phase consisted of potassium phosphate buffer (pH 3.0, 15 mM), acetonitrile and methanol (70:20:10, *v*/*v*/*v*), run at a flow rate of 0.8 mL/min. The method was validated according to international guidelines for specificity, linearity, precision, accuracy, and robustness.

### 2.5. ACAT-Se Content and Entrapment Efficiency

For the determination of the total ACAT-Se content in the NP suspension, the NP samples were diluted in acetonitrile (1:5, *v*/*v*), followed by vortex mixing for 10 min (1300 rpm) and ultrasound for 10 min at 40 °C. Then, the samples were diluted in mobile phase to 20 μg/mL, filtered through a 0.45 μm membrane, and injected into RP-LC system, using the previously described method. The total content of the active compound in the NP suspensions was calculated against a methanolic solution of ACAT-Se (3 mg/mL) diluted in mobile phase to the same concentration.

The entrapment efficiency (EE%) was determined by the ultrafiltration/centrifugation technique using Amicon Ultra-0.5 Centrifugal Filters (10,000 Da MWCO, Millipore, Cork, Ireland). An aliquot of the NP suspension was transferred to the centrifugal device and submitted to centrifugation at 3610× *g* for 20 min. The ultrafiltrate (free content) was analyzed by the RP-LC method. The EE% was calculated by the following equation:

$$EE\%=\left(\frac{Totalcontent-Freecontent}{Totalcontent}\right)\times 100\;$$

### 2.6. In Vitro Release Studies

The in vitro release studies of ACAT-Se from PLGA NPs were performed by the dialysis method. The samples (500 µL) were transferred to a dialysis bag (Sigma–Aldrich, 14,000 MWCO), sealed, and immersed in 50 mL of phosphate buffer solution (PBS), pH 7.4 or 5.4 (pH adjusted with NaOH or phosphoric acid, respectively), with constant shaking (150 rpm), in water bath at 37 °C for 24 h. At predetermined time intervals, 2 mL of the release medium was collected and replaced for an equivalent amount of fresh release medium to maintain the sink conditions. The collected samples were filtered through a 0.45 µm membrane and analyzed by the previously described RP-LC method. The release of free ACAT-Se (methanolic solution) was also evaluated in pH 7.4, for comparison proposes.

The Korsmeyer Peppas $\left(kt^{n}=\frac{Mt}{M\infty }\right)$ equation was used to understand the mechanisms by which the drug release happened. In this equation, *k* considers the geometric characteristics of the system, *n* gives the information about the diffusional release mechanism of a drug from a polymeric device and *Mt* and *M∞* are absolute values of drug released at time *t* and infinity [32,33].

### 2.7. In Vitro Antioxidant Activity

The percent scavenging activity of the NPs was determine using DPPH and ABTS assays [34,35]. NPs and free ACAT-Se were diluted at the concentrations of 25, 50, 100, 200 and 300 µg/mL. The samples (75 µL) were placed in 96-well plates with a 50 mM DDPH solution (150 µL) in methanol and kept in the in dark at room temperature for 30 min. The absorbance was measured at 550 nm using a microplate reader Multiskan FC (Thermo Fisher Scientific, Shanghai, China) (*Sample Abs*). The same procedure was used for the ABTS assay; the ABTS solution was prepared by mixing 5 mL of 7 mM ABTS in water with 88 µL 140 mM sodium persulfate, and this solution was kept in the dark at room temperature for 12 h. Subsequently, this solution was diluted in a 10 mM phosphate solution pH 7.0 to obtain a 42.7 µM of ABTS in the final solution. The absorbance was measured at 734 nm. The NPs turbidity interference (*Blank Abs*) was determinate preparing samples solutions with 150 µL of methanol or water instead of the DPPH or ABTS solutions, respectively. The negative control (*Negative control Abs*) was assessed by mixing the DPPH or ABTS solutions with 75 µL of water. Percent scavenging activity was calculated by the following equation:

$$Scavenging\;activity\;\%=\left(\frac{\left(Sample\;Abs-Blank\;Abs\right)\times 100}{Negative\;control\;Abs}\;\right)-100$$

### 2.8. In Vitro Cell Biocompatibility Studies

The study of the nonspecific cytotoxicity of NPs was assessed using the nontumor cell line 3T3 (murine Swiss albino fibroblasts), which was obtained from European Collection of Authenticated Cell Cultures (ECACC) repository by purchasing them at Sigma-Aldrich. The cells were cultured in DMEM medium (4.5 g/L glucose) supplemented with 10% (*v*/*v*) FBS, at 37 °C with 5% CO_2_. When they achieved approximately 80% confluence, were seeded (6.5 × 10^4^ cells/mL) in 96-well cell culture plates and incubated for 24 h. The cells were treated with free and nanoencapsulated ACAT-Se for 72 h at 37 °C with 5% CO_2_, and the cell viability was evaluated by the MTT assay using a microplate reader Multiskan FC (Thermo Fisher Scientific, Shanghai, China) set at 550 nm. The cell viability after treatment with the antitumor drugs methotrexate (MTX) (50 µg/mL) and DOX (10 µg/mL) was also assessed for comparison purposes.

### 2.9. Hemocompatibility Studies

The NPs hemocompatibility was evaluated using the hemolysis assay [22]. Erythrocytes were isolated from human blood, obtained from healthy volunteer donors invited according to the guidelines established by the Ethics Committee in Research, from the Federal University of Santa Maria, Brazil (protocol CAAE 44017921.3.0000.5346). Red blood cells were isolated, by centrifugation, washed and suspended in isotonic PBS (pH 7.4; 300 mOsmoL/L) at a cell density of 8 × 10^9^ cell/mL. The ACAT-Se-PLGA-77KL-NPs and PLGA-77KL-NPs were diluted in PBS pH 7.4 at concentrations of 5, 7.5, and 10% (*v*/*v*) corresponding to 150, 225 and 300 µg/mL of ACAT-Se, respectively. Free ACAT-Se was also assessed in the same concentrations for comparison purposes. The samples were incubated with 25 µL of the erythrocyte suspension for 5 h under gentle shaking and then centrifuged at 10,000× *g* for 5 min to stop the reaction. Positive and negative controls were prepared by incubating the erythrocyte suspension with water and PBS, respectively. Afterwards, an aliquot of 200 µL of at sample were placed in 96-well plates and the absorbance was determined at 550 nm using a microplate reader Multiskan FC (Thermo Fisher Scientific, Shanghai, China).

### 2.10. pH-Dependent Membrane-Lytic Activity of Nanoparticle

The hemolysis assay was used to determine the pH-dependent membrane-lytic activity of the NPs using the erythrocytes as a model of endosomal membrane [36]. An aliquot of 25 µL of erythrocytes suspension, prepared as described for the hemocompatibility assay, was incubated with the NP suspensions diluted in PBS pH 7.4, 6.6 or 5.4 at the concentrations of 5, 7.5 and 10% (*v*/*v*), corresponding to 150, 225 and 300 µg/mL of ACAT-Se, respectively. For the positive control, the erythrocytes suspension was diluted in water, and for the negative control in PBS pH 7.4, 6.6 or 5.4. The samples were incubated at room temperature under constant shaking for 5 h and then centrifuged at 10,000× *g* for 5 min. Supernatants were placed in 96-well plates and the absorbance was determined at 550 nm using a microplate reader Multiskan FC (Thermo Fisher Scientific, Shanghai, China).

### 2.11. In Vitro Protein Corona

The NP suspensions were dispersed in cell culture medium (DMEM 5% FBS) or in plasma [37]. The mean hydrodynamic diameter was determined using a Malvern Zetasizer ZS (Malvern Instruments, Malvern, UK), immediately after dilution (*t* = 0 h) and after 24 and 72 h of incubation at 37 °C, simulating the environment found during the in vitro cytotoxicity experiments or in vivo conditions.

### 2.12. In Vitro Antitumor Activity

The MCF-7 (human breast cancer) cell line was obtained from Eucellbank of Celltec UB (University of Barcelona, Spain) and cultured in DMEM medium (4.5 g/L glucose) supplemented with 10% (*v*/*v*) FBS, at 37 °C with 5% CO_2_. The cells were seeded into 75 cm^2^ cultivation flasks and harvested using trypsin-EDTA when the cells reached approximately 80% confluence. The multidrug-resistant (MDR) cell line NCI/ADR-RES (human ovarian cancer cells) was kindly donated by Dr. Antoni Benito from the University of Girona (Spain) and cultured continuously in the same DMEM medium (4.5 g/L glucose), but containing doxorubicin (DOX) 1 µg/mL.

Both tumor cell lines were used to evaluate the potential in vitro antineoplastic activity of the free and nanoencapsulated ACAT-Se. The MCF-7 and NCI/ADR-Res cells (1 × 10^5^ cells/mL) were seeded in 96-well cell culture plates in 100 μL of complete culture medium, incubated for 24 h. Then, the medium was replaced for 100 μL of the treatments diluted in DMEM medium with 5% FBS. The free ACAT-Se and ACAT-Se-PLGA-77KL-NPs were prepared at the concentrations of 0.5, 5, 10, 20, 40 and 60 µg/mL of ACAT-Se. The PLGA-77KL-NPs were diluted at the same proportion of the ACAT-Se NPs to evaluate the interference of the NP matrix. Control cells were exposed to the DMEM medium with 5% FBS only. Additionally, the antitumor drugs MTX (50 µg/mL) and DOX (10 µg/mL) were used as positive controls. Cells were incubated for 24 or 72 h and their viability was assessed by the MTT and NRU assays. For the MTT assay the medium was replaced by 100 µL of 0.5 mg/mL MTT solution in DMEM without FBS, incubated of 3 h at 37 °C with 5% CO_2_. Then, the medium was replaced by 100 µL of DMSO to dissolve the purple formazan product. Similarly, for the NRU assay, the medium was discarded and 100 µL of 50 µg/mL NR dye solution was added, incubated of 3 h at 37 °C with 5% CO_2_. After the incubation time, the microplates were washed with PBS, then the PBS was replaced by 100 µL of a solution containing 50% ethanol and 1% acetic acid in distilled water. For both assays, the microplates were shaking for 10 min at room temperature and the absorbance was measured at 550 nm using a microplate reader Multiskan FC (Thermo Fisher Scientific, Shanghai, China).

### 2.13. Synergic In Vitro Antitumor Activity with Doxorubicin

This assay was applied to study the effect of the interaction between the free or nanoencapsulated ACAT-Se and the reference chemotherapeutic drug DOX. The study was also performed comparatively on the tumor cell lines MCF-7 and NCI/ADR-RES, and the treatments were prepared at the concentrations of 10, 20, 40 and 60 µg/mL of ACAT-Se with fixed DOX concentration of 0.05 and 10 µg/mL for MCF-7 and NCI/ADR-RES, respectively. The synergic activity of PLGA-77KL-NPs (blank NPs) was also evaluated for comparison purposes. They were diluted at the same proportion of the ACAT-Se NPs and incubated with the same DOX concentrations. After 72 h of incubation, cell viability was assessed using MTT and NRU assays. Results were used to calculate the combination index (CI) and dose-reduction index (DRI) by the median-effect method using CompuSyn software (ComboSyn, Inc., Paramus, NJ, USA). According to Chou–Talalay method, CI < 0.9, 0.9 < CI > 1.1, and CI > 1.1 indicate synergism, additive effect, and antagonism, respectively [1,38,39,40]. The DRI is a measure of how many fold the dose of each drug in a synergistic combination may be reduced at a given effect level, compared with the doses of each drug alone. DRI > 1 indicate a favorable dose reduction and greater DRI value indicates a greater dose reduction effect [38,41].

### 2.14. Statistics

Results are expressed as mean ± standard error (SE) or mean ± standard deviation (SD) and statistical analyses were performed using one-way analysis of variance (ANOVA) to determine the differences between the datasets, followed by Student–Newman–Keuls test for multiple comparisons, using SPSS^®^ software (SPSS Inc., Chicago, IL, USA). The robustness of the RP-LC method was verified using Minitab 17 (MINITAB^®^ Statistical Software, Release 17, Minitab Inc., State College, PA, USA). All experiments were performed at least three times. *p* < 0.05 was considered to be significant.

## 3. Results

### 3.1. Characterization of Nanoparticles

The results of the determination of mean hydrodynamic diameter, PDI, ZP and pH of the NPs with and without ACAT-Se are showed in Table 1. The NP suspensions presented nanometric size (~120 nm), low PDI values that indicates a homogenous size distribution, negative zeta potential (~−4 mV) and neutral/slightly acidic pH. No significant difference was observed between the NPs with or without ACAT-Se (*p* > 0.05).

### 3.2. Analytical Method

The developed RP-LC method was linear in the range 1–40 µg/mL (y = 47,589.60 × − 5772.05, *r* = 0.9998), showing significant linear regression (F_calculated_ = 15,339 > F_critical_ = 4.8, *p* < 0.05) with no linearity derivation (F_calculated_ = 1.5 < F_critical_ = 3.3, *p* > 0.05). Limit of detection (0.0056 µg/mL) and the limit of quantification (0.17 µg/mL) were obtained using the signal-to-noise ratio. The relative standard deviation values for repeatability, interday, and between-analyst precision lower than the acceptance criterion of 2% proved the method precision. The recovery test was used to evaluate the accuracy, evidenced by recovery values between 98 and 102%. Method specificity was proved by a peak-purity evaluation using the PDA detector, ACAT-Se peak showed peak purity index higher than 0.9999 confirming the absence of any co-eluting interference. Finally, robustness was evaluated using a factorial model with three factors in two levels (flow rate ± 2%, organic solvent ± 2% and buffer solution pH ± 0.2). Pareto chart indicates that assay values are not affected by small modifications in experimental environment (*p* > 0.05), confirming the method robustness.

### 3.3. ACAT-Se Content and Entrapment Efficiency

The previously described RP-LC method was successfully applied to determine the ACAT-Se content into the NPs, which was found as 2.9 ± 0.19 mg/mL. The EE was determined as 64.1 ± 2.3% (Table 1).

### 3.4. In Vitro Release Studies

**Figure 1:**
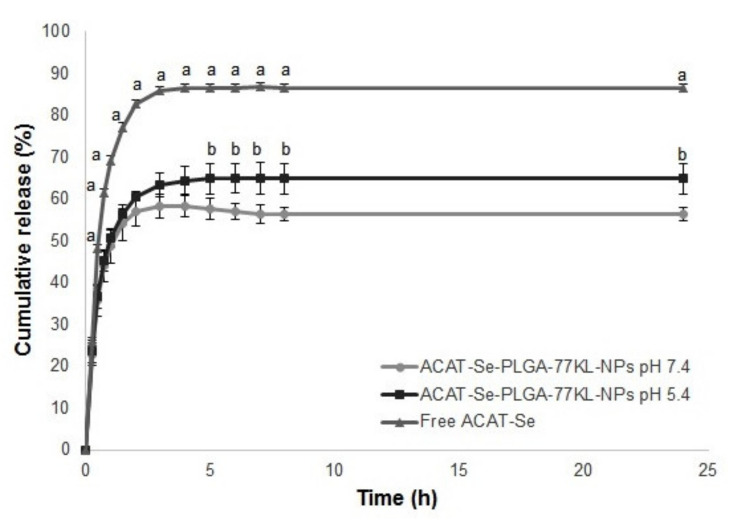
In vitro release of free and nanoencapsulated ACAT-Se. Results are expressed as mean ± SE of three independent experiments. Statistical analyses were performed using ANOVA, followed by Student–Newman–Keuls multiple comparison test. ^a^ Significant difference between Free ACAT-Se and ACAT-Se-PLGA-77KL-NPs *(p* < 0.05), ^b^ between PLGA-77KL-NPs pH 7.4 and 5.4 (*p* < 0.05).

The cumulative release of ACAT-Se is shown in Figure 1. After 24 h, 56.3 ± 1.5% and 64.9 ± 3.0% of ACAT-Se was release from the NPs in pH 7.4 and 5.4, respectively, while 86.6 ± 0.88% of free ACAT-Se was released. By *n* values of the Korsmeyer Peppas equation, the release mechanism of ACAT-Se from the NPs is Fickian diffusion (*n* = 0.26 and 0.40 in pH 7.4 and 5.4, respectively).

### 3.5. In Vitro Antioxidant Activity

**Figure 2:**
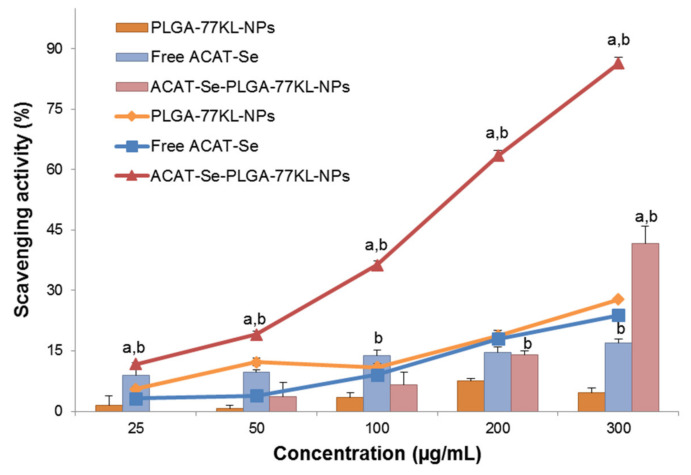
Scavenging activity of free ACAT-Se, PLGA-77KL-NPs and ACAT-Se-PLGA-77KL-NPs using ABTS (line graph) and DPPH (bar graph) assays. Results are expressed as mean ± SE of three independent experiments. Statistical analyses were performed using ANOVA followed by Student–Newman–Keuls multiple comparison test. ^a^ Significantly different from Free ACAT-Se (*p* < 0.05), ^b^ from PLGA-77KL-NPs (*p* < 0.05).

The scavenging activity of free ACAT-Se, PLGA-77KL-NPs, and ACAT-Se-PLGA-77KL-NPs is presented is Figure 2. In DPPH assay, free ACAT-Se demonstrate low scavenging activity (8.9–16.9%); however, the nanoencapsulated form significantly increase this activity at 300 µg/mL (41.6 ± 4.5%, *p* < 0.05). On the other hand, ABTS assay was more sensitive to detect differences between free and nanoencapsulated ACAT-Se radical scavenging activity. The ACAT-Se-PLGA-77KL-NPs has superior scavenging activity than free ACAT-Se in all the concentrations tested (*p* < 0.05).

### 3.6. In Vitro Cell Biocompatibility Studies

**Figure 3:**
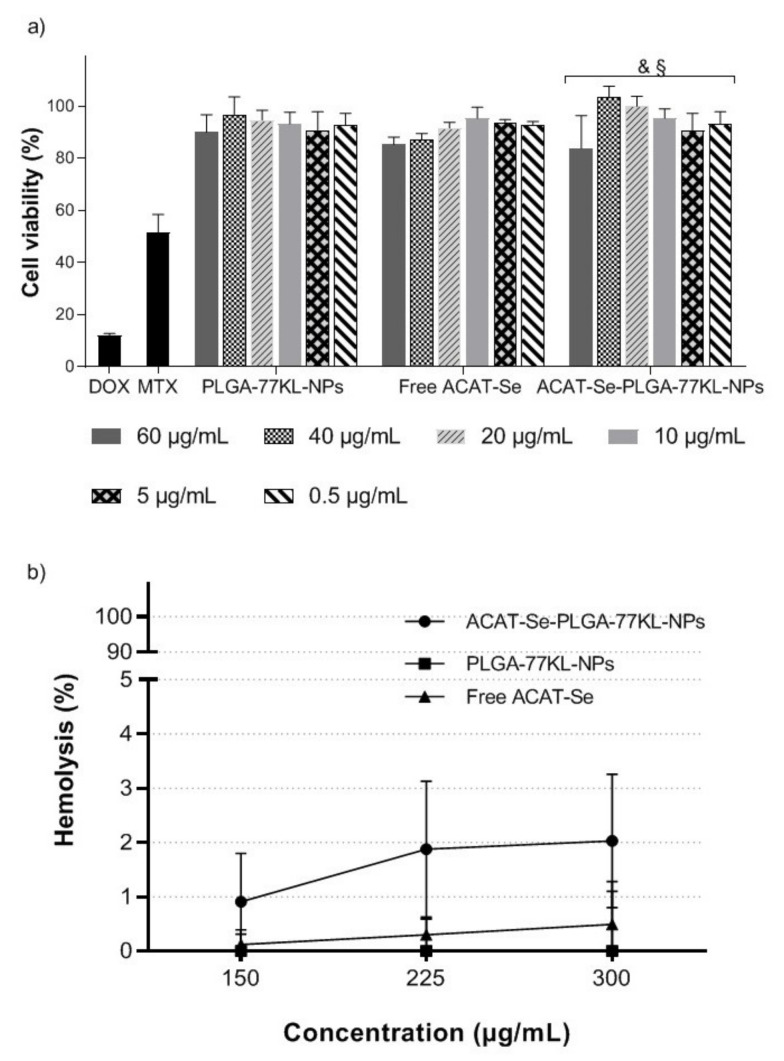
(**a**) In vitro cell biocompatibility of NPs evaluated using nontumor cell line 3T3 by MTT assay. MTX (50 µg/mL) and DOX (10 µg/mL) were also assessed for comparison purposes. Statistical analyses were performed using ANOVA followed by Student–Newman–Keuls multiple comparison test. ^&^ Significant difference from MTX (*p* < 0.05) and ^§^ significant difference from DOX (*p* < 0.05). (**b**) Hemocompatibility study after 5 h of incubation with human erythrocytes. Each value represents mean ± SE of three experiments.

Free ACAT-Se, ACAT-Se-PLGA-77KL-NPs, and PLGA-77KL-NPs showed no cytotoxic effects towards the nontumor cell line 3T3 after 72 h of treatment. Furthermore, the ACAT-Se NPs were less cytotoxic (cell viability between 83.8 and 99.8%) than DOX and MTX (cell viability of 11.9 and 51.4%, respectively, *p* < 0.05) (Figure 3a).

### 3.7. Hemocompatibility Studies

Blood compatibility of the NP suspension was evaluated by the hemolysis assay, which estimates the erythrocyte-damage by the quantification of the hemoglobin released. After 5 h of incubation, NP suspensions and the free compound were nonhemolytic, as the hemolysis rate was lower than 5% in all tested conditions (maximum hemolysis rate of 2.0 ± 1.2% for ACAT-Se-PLGA-77KL-NP) (Figure 3b).

### 3.8. pH-Dependent Membrane-Lytic Activity of Nanoparticle

**Figure 4:**
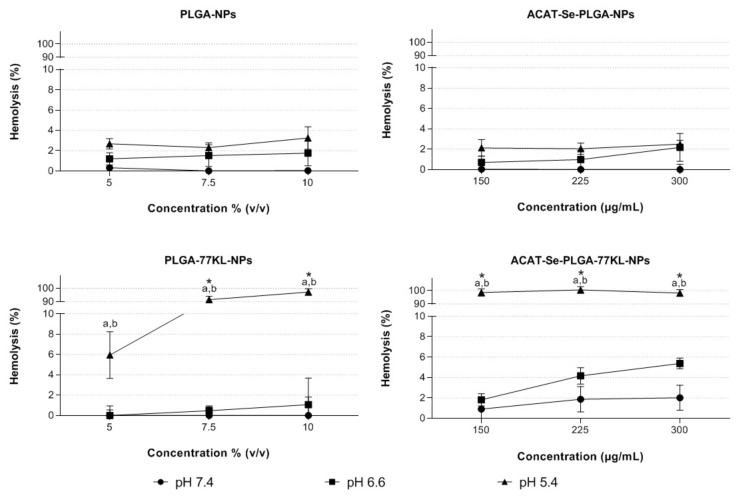
pH-dependent membrane-lytic activity of NPs after 5 h of incubation with human erythrocytes at different pH values. Each value represents mean ± SE of three experiments. Statistical analyses were performed using ANOVA followed by Student–Newman–Keuls multiple comparison test. a Significantly different from pH 7.4 (*p* < 0.05) and b from pH 6.6 (*p* < 0.05). Asterisk indicates significant difference between NPs with and without 77KL (*p* < 0.05).

In this assay, the erythrocytes were used as a model of endosomal membrane to verify if the 77KL confers to the NPs the ability to disrupt lipid bilayer membranes in a pH-dependent manner. No significant hemolysis rates were obtained for the NP without ACAT-Se and 77KL under all tested conditions (Figure 4). On the other hand, the presence of 77KL on the PLGA-77KL-NPs increased significantly (*p* < 0.05) the hemolysis at pH 5.4 to 97.1%, in comparison to 1.8 and 0.22% at pH 6.6 and 7.4, respectively, at concentration of 10% (*v*/*v*). In addition, at pH 5.4 the PLGA-77KL-NPs were 39.7 and 29.8-fold more hemolytic than PLGA-NPs at the concentrations of 7.5 and 10%, respectively.

Likewise, the ACAT-Se NPs presented similar results to those observed for the NPs without ACAT-Se. ACAT-Se-PLGA-NPs did not generate hemolysis on the tested conditions. In contrast, the ACAT-Se-PLGA-77KL-NPs presented low hemolysis at physiological pH (between 0.91 and 2.0%), a slight increase was observed at pH 6.6 (between 1.8 and 5.4%), and the membrane-lytic activity increased significantly at pH 5.4 (*p* < 0.05) in all tested concentrations (hemolysis between 98.2 and 100%). Furthermore, at pH 5.4 the ACAT-Se-PLGA-77KL-NPs were 46.4, 49.2 and 39.4-fold more membranolytic than the NPs without 77KL (*p* < 0.05), at 150, 225 and 300 µg/mL, respectively. Finally, the pH-dependent activity of nonencapsulated ACAT-Se was assessed, but negligible hemolysis rates were observed in the tested conditions.

### 3.9. In Vitro Protein Corona

**Figure 5:**
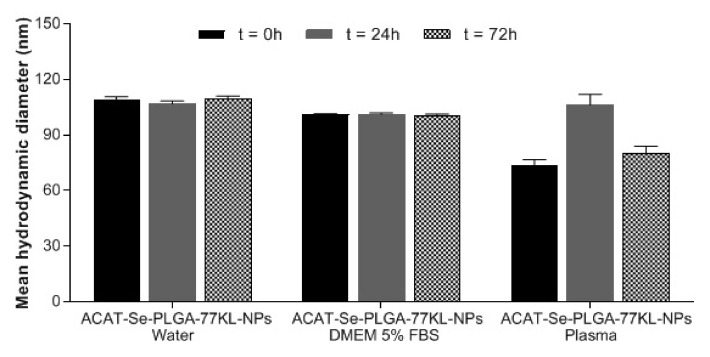
Mean hydrodynamic diameter of ACAT-Se-PLGA-77KL-NPs after 0, 24 and 72 h of incubation with water, DMEM 5% FBS and plasma. Each value represents mean ± SE of three experiments. Statistical analyses were performed by Student *t*-test and no significant differences were found.

The NPs did not show increase in mean hydrodynamic diameter (*p* > 0.05) after incubation at 37 °C with human plasma or cell culture medium (DMEM with 5% FBS) during 72 h (Figure 5). These results indicated that the proteins of plasma or culture medium did not bind on the NP surface. A decrease in the NP mean hydrodynamic diameter was observed after incubation with plasma at 0 and 72 h; however, the statistical analysis evidenced that the observed variations in the mean size were not statistically significant.

### 3.10. In Vitro Antitumor Activity

**Figure 6:**
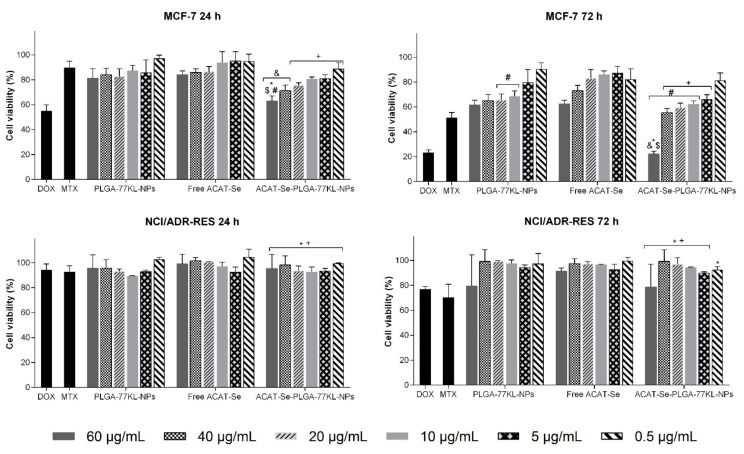
In vitro cell viability by MTT assay in MCF-7 and NCI/ADR-RES cell lines after 24 and 72 h of treatment. antitumor drugs MTX (50 µg/mL) and DOX (10 µg/mL) were used as positive controls. Data are expressed as mean of three independent experiments ± SE. Statistical analyses were performed using ANOVA followed by Student–Newman–Keuls multiple comparison test. ^$^ Significant difference from PLGA-77KL-NPs (*p* < 0.05), ^#^ significant difference from free ACAT-Se (*p* < 0.05), ^&^ significant difference from MTX (*p* < 0.05), * no significant difference from DOX (*p* > 0.05), and ^+^ no significant difference from MTX (*p* > 0.05).

The cytotoxicity of free ACAT-Se or nanoencapsulated ACAT-Se was evaluated against a sensitive and a resistant tumor cell line, MCF-7 and NCI/ADR-RES, respectively. Firstly, by MTT assay (Figure 6), the results evidenced that the antitumor activity of ACAT-Se-PLGA-77KL-NPs against MCF-7 cells was greater than that of the free compound, especially after 72 h of treatment. Moreover, at 60 µg/mL the NPs were more cytotoxic than MTX, while no difference was observed in comparison to DOX cytotoxicity, after 24 and 72 h of incubation. In contrast, the nanoencapsulation of ACAT-Se did not increase its cytotoxicity toward the resistant cell line; however, the ACAT-Se-PLGA-77KL-NPs presented cytotoxicity equivalent to the positive controls DOX and MTX in almost all the tested conditions.

**Figure 7:**
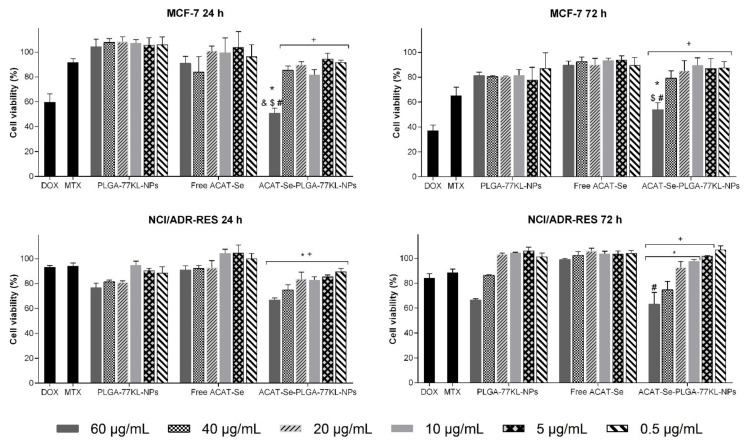
In vitro cell viability by NRU assay in MCF-7 and NCI/ADR-RES cell lines after 24 and 72 h of treatment. antitumor drugs MTX (50 µg/mL) and DOX (10 µg/mL) were used as positive controls. Data are expressed as mean of three independent experiments ± SE. Statistical analyses were performed using ANOVA followed by Student–Newman–Keuls multiple comparison test. ^$^ Significant difference from PLGA-77KL-NPs (*p* < 0.05), ^#^ significant difference from free ACAT-Se (*p* < 0.05), ^&^ significant difference from MTX (*p* < 0.05), * no significant difference from DOX (*p* > 0.05), and ^+^ no significant difference from MTX (*p* > 0.05).

The NRU assay (Figure 7) was less sensitive than MTT to detect the cytotoxic effects on MCF-7 cells; nevertheless, it was evidenced that the cell viability decreased from 91.1% for free ACAT-Se to 50.9% for ACAT-Se-PLGA-77KL-NPs (5.48-fold more cytotoxic, *p* < 0.05) after 24 h at the higher tested concentration. Conversely, NRU assay was more sensitive than MTT to detect the cytotoxicity on NCI/ADR-RES cells. Through this endpoint, the cell viability after 72 h incubation with the nanoencapsulated ACAT-Se at 60 µg/mL was 63.3%, lower than that detected by the MTT assay (78.8%). At this same condition, it was also evidenced that the ACAT-Se-PLGA-77KL-NPs were more cytotoxic than the non-encapsulated ACAT-Se (cell viability of 99.2%, *p* < 0.05).

### 3.11. Synergic In Vitro Antitumor Activity with Doxorubicin

**Figure 8:**
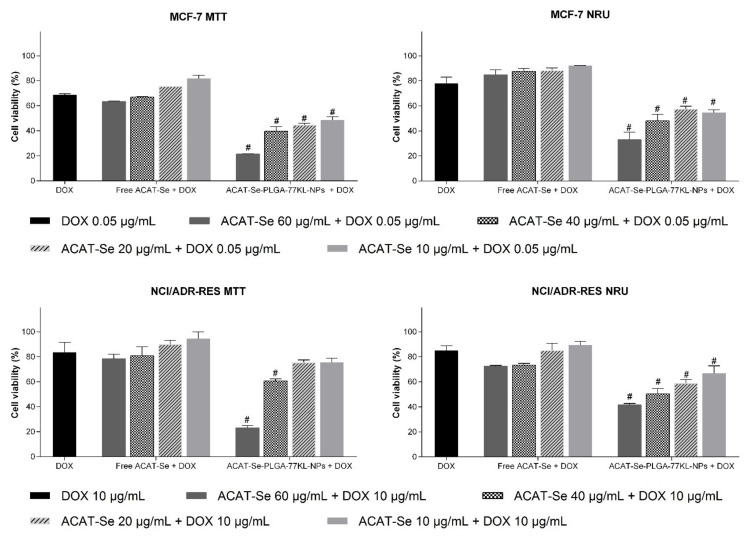
In vitro cell viability as detected by MTT and NRU assays after 72 h coincubation of free or nanoencapsulated ACAT-Se with DOX in MCF-7 and NCI/ADR-Res cell lines. Data are expressed as mean of three independent experiments ± SE. Statistical analyses were performed using ANOVA followed by Student–Newman–Keuls multiple comparison test. ^#^ Significant difference from free ACAT-Se + DOX (*p* < 0.05).

**Figure 9:**
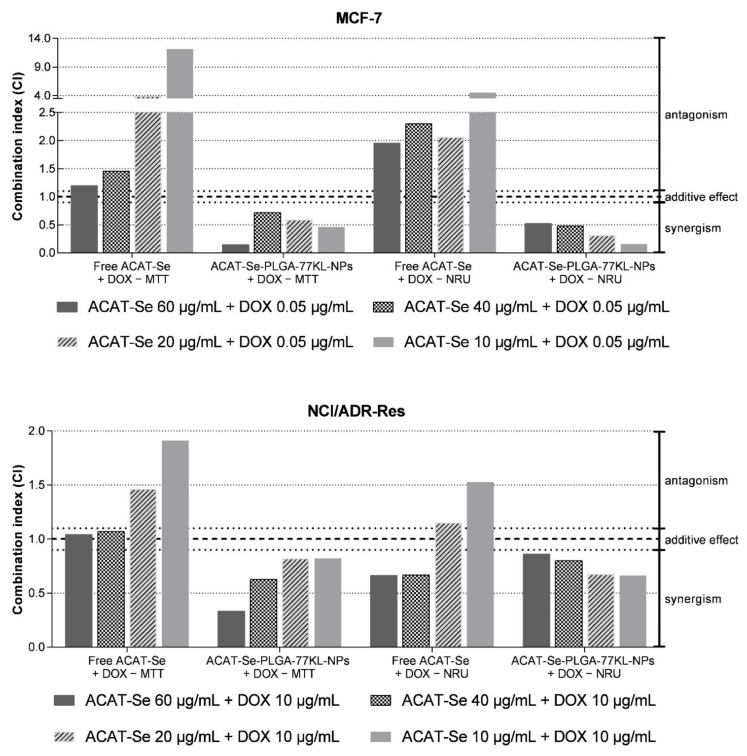
Combination index values for association of free ACAT-Se or ACAT-Se-PLGA-77KL-NPs with DOX in MCF-7 and NCI/ADR-Res cell lines.

The cytotoxic effects of ACAT-Se-PLGA-77KL-NPs after coincubation with DOX were greater than those achieved with free ACAT-Se coincubated with DOX in almost all tested concentrations. On NCI-ADR/RES and MCF-7 cells by both viability assays (Figure 8). Noteworthy, in MCF-7 cell line the combination of NPs with DOX cause synergistic effect in all concentrations as detected by MTT and NRU assays (CI < 0.9) (Figure 9). Additionally, by the CI values observed, this association can be consider a strong synergism in some concentrations (CI = 0.1453 at 60 µg/mL ACAT-Se + 0.05 µg/mL DOX, by MTT assay and CI = 0.1578 at 10 µg/mL ACAT-Se + 0.05 µg/mL DOX, by NRU assay) [1]. In contrast, the association of free ACAT-Se with DOX resulted in antagonism (CI > 1.1) in MCF-7 cells at all tested concentrations as detected by both viability assays.

Synergistic interactions were also evidenced for ACAT-Se-PLGA-77KL-NPs after coincubation with DOX in the resistant cell line in all tested concentrations, as detected by MTT and NRU assay (CI < 0.9). In contrast, the treatment with free ACAT-Se associated with DOX cause additive effect (40 and 60 µg/mL of ACAT-Se) or antagonism (10 and 20 µg/mL of ACAT-Se) by the MTT assay, and synergism (40 and 60 µg/mL of ACAT-Se) or antagonism (10 and 20 µg/mL of ACAT-Se) by NRU assay.

Finally, the association of ACAT-Se-PLGA-77KL-NPs and DOX in all conditions resulted in DRI values >1, suggesting that this combination therapy is able to reduce the dose of each individual treatment.

The association of PLGA-77KL-NPs (blank NPs without the organoselenium compound) and DOX resulted in low cytotoxic effects in both MCF-7 (cell viability between 74–80% and 79–83%, by MTT and NRU assay, respectively) and NCI/ADR-RES cells (cell viability between 83–87% and 69–79%, by MTT and NRU assay, respectively) (Figure S1, Supplementary Materials). Moreover, the CI values indicated that this association results in antagonism (CI > 1.1) in MCF-7 cells, by both MTT and NRU assays, in all tested concentrations. Likewise, in NCI/ADR-RES cells, the association of PLGA-77KL-NPs and DOX displayed antagonism by MTT assay and antagonism (40 and 60 µg/mL) or additive effect (10 and 20 µg/mL) by NRU assay (Figure S2, Supplementary Materials).

## 4. Discussion

Chemotherapy is considered one of the most significant cancer treatment approaches; however, chemotherapeutic agents are nonspecific, attacks both normal and cancerous cells, leading to severe toxicity to normal tissues [42,43]. Polymeric NPs have great potential to improve the efficacy of cancer treatment by their ability to accumulate at tumor sites [17,43]. Additionally, a popular approach to target cancer cells is the exploration of pH difference between healthy (~7.4) and tumoral tissues (6.5–7.2) [44]. Therefore, we design promising pH-sensitive PLGA-based NPs encapsulating the organoselenium compound ACAT-Se as a new alternative to antineoplastic therapy. For this purpose, the NPs were modified by the incorporation of the pH-sensitive surfactant 77KL, and the surfactant Pluronic^®^ F-127 was utilized to stabilize the NPs, due its ability to overcome MDR in cancer cells and also due its higher cytocompatibility among some other poloxamers [27,45].

The NP suspensions were successfully prepared by the nanoprecipitation method, which is widely used due to its simplicity, quickness, reproducibility, and safety [46,47]. The developed NPs appeared macroscopically as a homogeneous slightly white (PLGA-77KL-NPs) or creamy-white (ACAT-Se-PLGA-77KL-NPs) solution. The hydrodynamic size was found to be between 100 and 200 nm, which is consider an optimal size for drug delivery systems since the NPs take the advantage of EPR effect in tumors and avoid filtration in the spleen and the uptake in the liver [48]. The zeta potential provides a indicative evidence towards the nature of surface charge of the NPs [49]. The developed NPs showed negative ZP that can be attributed to the carboxyl groups of PLGA residing on the surface of the NPs [50]; conversely, the low module value can be associated to the shielding effect of the coated nonionic surfactants (Pluronic^®^ F-127 and Span^®^ 80) present on the surface of NPs [51]. Since zeta potential value can indicate colloid stability via electrostatic repulsions, it does not provide any insight on the van der Waals forces. Therefore, it is not uncommon to come across stable colloids with ZP between −30 and +30 mV, and vice versa [49]. Moreover, Honary & Zahir [52] suggest that the performance of ZP measurements in distilled water rather than in physiological salt solution is the best way to determine the physical stability of the NPs. Therefore, this measuring condition could be a complementary experiment to corroborate our results.

In addition, the total ACAT-Se content in the NP suspension and the EE was successfully determinate by the developed RP-LC method. This method was also able to measure the amount of ACAT-Se released from the NPs. Moreover, the Korsmeyer–Peppas model indicates that the release of ACAT-Se from the NPs follows the Fickian diffusion release mechanism, the same mechanism was report for others PLGA NPs [20,53,54].

Organochalcogenium compounds were reported to be effective against free radical species and presented exciting results as radical scavengers [9,55]. In this sense, we evaluate the antioxidant potential of the free and nanoencapsulated ACAT-Se using DPPH and ABTS assays, both based on absorption decreases upon exposure to proton radical scavengers [56]. Free ACAT-Se presented poor scavenging activity using the DPPH assay, and this result corroborates with the earlier study reported by da Rosa and coworkers, who showed that free ACAT-Se was ineffective in scavenging the DPPH free radical at 1 mM (381 µg/mL). On the other hand, the nanoencapsulation increased the radical scavenging activity of ACAT-Se 2.5-fold (at 300 µg/mL). This same tendency was observed in ABTS assay, in which the ACAT-Se-PLGA-77KL-NPs presented superior radical scavenging activity in comparison to free ACAT-Se. It is worth mentioning that the increase of the antioxidant activity by the nanoencapsulation was also reported in other studies [57,58,59,60]. Some authors suggest that the increase of this activity could be attributed to the superior contact surface area between the H donator and DPPH or ABTS molecules provided by the nanometric size of the particles, which thus offers easier access of the hydrogen atom to the radical site [58,60]. Moreover, the higher sensitivity of ABTS assay can be associated to its faster reaction kinetics and higher response to antioxidants [56].

The pH-dependent hemolysis assay evidenced that the inclusion of the 77KL in the NP suspensions confer to them a pH-dependent behavior. Moreover, this behavior did not change after the entrapment of ACAT-Se into NPs. The increase hemolysis at pH 5.4 could be explained by a modification in the hydrophobic/hydrophilic balance of 77KL by the protonation of it carboxylic group, which results in an increase of its hydrophobicity, causing membrane solubilization or altering the permeability of the membrane, hence, inducing cell lysis [21,61]. This remarkable pH-responsive behavior suggests the potentiality of these NPs as an effective nanocarrier for intracellular drug delivery. Finally, these results corroborated our previous studies, in which the NPs with 77 KL or 77 KS (surfactant with sodium counterion) showed increased membrane-lytic activity in the pH range characteristic of endosomal compartments [19,20,21,22].

Considering that is no available regulatory guidelines for the evaluation of toxicity of nanoparticulate materials, the use of in vitro assays are highly important [62]. In this context, the hemocompatibility of the developed NPs was evaluated by the hemolysis assay. This study assess the impact of physicochemical characteristics of NPs (size, porosity and surface functionality) on red blood cells [62]. The NP suspensions presented nonhemolytic activity regardless of the tested concentration; these results suggest that the NPs are hemocompatible. Furthermore, we evaluate the NPs cytotoxicity using a nontumor cell model. Here, we found no significant cytotoxic effects after cell treatment with free and nanoencapsulated ACAT-Se for 72 h, suggesting the biocompatibility of our proposed nanosystem.

The study of protein corona formation is an important tool to predict NPs behavior in biological systems [63]. Our results indicate that the plasma or cell culture medium proteins did not bind to the ACAT-Se-PLGA-77KL-NPs surface. In this regard, low protein adsorption is interesting, since protein corona might activate immune cells, promoting phagocytosis that resulting in NPs clearance [64].

Considering that ACAT-Se is an thymidine analogue, we suggest that its cytotoxicity can be related to inhibition of intracellular enzymes (like polymerases or ribonucleotide reductase), or to inhibition of DNA chain elongation, by the assimilation of its active form in DNA chain [9,65]. Cell viability studies were performed using MTT and NRU assays. The MTT assay measure mitochondrial function, by the conversion of yellow tetrazolium salt to a purple formazan crystal through the action of mitochondrial dehydrogenases. While NRU assay measure the functionality of the lysosomes and cell membranes by the accumulation of NR dye in the lysosomes of viable cells [22,66]. In MCF-7 cells, the two endpoints evidenced improve on the antitumor activity of ACAT-Se with its association to the NPs. In contrast, on NCI/ADR-RES cells the benefits of nanoencapsulation were only observed by the NRU assay. In this case, the ACAT-Se-PLGA-77KL-NPs seems to change the functionality of the lysosomes and/or cell membranes of the MDR cells with no important disorders in mitochondrial function. Consequently, the different selectivity of the two endpoints on MCF-7 and NCI/ADR-RES cells suggests that the nanocarrier interact by different mechanisms in each cell type. In addition, resistant/MDR cell lines present diverse resistance mechanisms, such as increased activity of drug efflux pump, decreased drug influx, activation of DNA repair and metabolic modifications [67,68]. Therefore, these mechanisms might be responsible for the low cytotoxic activity of all monotherapies tested by MTT assay in our study. However, the results achieved in the synergism assay evidenced that the combination of ACAT-Se-PLGA-77KL-NPs and DOX was able to sensitize the MDR cell line, suggesting that this association can overcome the resistance mechanisms of NCI/ADR-RES cells. Finally, in the same concentrations that the NPs are cytotoxic to the tumor cells, no cytotoxic effects were evidenced in the nontumor cell line. Therefore, these results suggest that the NPs present high selectivity for the tumor cells.

The PLGA-77KL-NPs (blank NPs) present a slight cytotoxicity in MCF-7 cells as detected by the MTT assay, especially after 72 h of treatment. The cytotoxic effects of this nanoformulation can be associated with the NP size, shape and components (especially the surfactants), which might interfere within the cell membrane integrity, resulting in an imbalance between intra and extracellular ions, proteins and vital molecules required to keep the normal cell functions [69,70]. Nevertheless, the cytotoxicity displayed by the unloaded-NPs are expressively lower than that revealed by ACAT-Se-PLGA-77KL-NPs, indicating, thus, that the antiproliferative activity of the latter is majority related to the presence of ACAT-Se and not due to the NP matrix.

Combining two or more therapeutic agents is an effective approach to improve the efficacy of an antitumor treatment. By the combination therapy, it is possible to target multiple pathways, which can allow the reduction of tumor growth, metastatic potential, and drug resistance, thus increasing patient survival rate [1,71]. Furthermore, combination therapy has the potential to decrease treatment toxicity, allowing use a lower dosage of each individual drug. Therefore, this approach can increase the chance of disease control and decrease the chance of cancer cells becoming increasingly malignant and incurable [71]. Moreover, different selenium compounds present synergistic activity with chemotherapeutic drugs on tumor cell lines [1,72,73,74]. In this context, we decided to study the antitumor effects of the association of free or nanoencapsulated ACAT-Se with the antitumor drug DOX. Besides that, as ACAT-Se has antioxidant activity, especially after nanoencapsulation, its association with DOX can also be interesting to reduce the side effects induced by this drug, since reactive oxygen species (ROS) apparently plays a key role in doxorubicin-induced cardiotoxicity [3,30].

Our data revealed that free ACAT-Se and PLGA-77KL-NPs in combination with DOX were mostly antagonistic. Conversely, the combination treatment of ACAT-Se-PLGA-77KL-NPs and DOX resulted in synergistic effect (CI < 0.9), indicating that it is possible to reduce the concentrations of each individual treatment (DRI > 1) in both cell lines. Therefore, our overall results evidenced that the synergistic effect is actually attributed to the association of DOX and ACAT-Se nanoencapsulated into the NPs, since we did not observe synergism by the association of free ACAT-Se and blank PLGA-77KL-NPs with DOX. In addition, the combination of ACAT-Se-PLGA-77KL-NPs and DOX was the only condition able to sensitize the resistant MDR tumor cells, achieving 76.7% of cytotoxicity as detected by the MTT assay. The synergistic effect of the combined therapy with the NPs and DOX in the resistant cell line can be also associated to the Pluronic^®^ F-127 ability to inhibit P-gp efflux pumps, since over expression of P-gp is one of the resistance mechanisms in MDR cells [75,76]. These results suggest that the association of the proposed pH-responsive nanocarrier with DOX is a promising approach to improve antitumor therapy and overcome MDR.

## 5. Conclusions

In this study, we successfully prepared pH-sensitive PLGA-NPs loaded with the organoselenium compound ACAT-Se. The pH-dependent membrane-lytic study evidenced that the inclusion of 77KL in the NPs gives it a pH-dependent behavior. Nanoencapsulation of ACAT-Se was able to increase its antioxidant potential. Moreover, ACAT-Se-PLGA-77KL-NPs displayed higher in vitro antitumor activity than free ACAT-Se in MCF-7 cells. In addition, this formulation displayed negligible cytotoxicity to the nontumor cell line 3T3, highlighting its selectivity to tumor cells. Hemocompatibility of the NPs was evidenced by the hemolysis assay. Finally, the nanoformulation proposed in this study presented synergistic antitumor activity with DOX, and by this approach, it was possible to sensitize NCI/ADR-RES cells overcoming MDR. Based on the overall results, the ACAT-Se-loaded nanocarrier system can be proposed as a promising approach to overcome MDR, besides its great potentiality to improve the individual and/or combined antitumor therapy.

## Figures and Tables

**Table 1:** Physicochemical characterization of NP suspensions.

	Particle Size (nm) ± SD	PDI ± SD	ZP (mV) ± SD	pH ± SD	ACAT-Se Content (mg/mL) ± SD	Entrapment Efficiency (%) ± SD
ACAT-Se-PLGA-77KL-NPs	118.9 ± 11.4	0.129 ± 0.04	−4.3 ± 0.70	7.1 ± 0.78	2.9 ± 0.19	64.1 ± 2.3
PLGA-77KL-NPs	126.5 ± 10.8	0.136 ± 0.02	−3.4 ± 0.67	6.6 ± 0.89	-	-

SD, standard deviation, *n* = 3.

## Supplementary Materials

The following supporting information can be downloaded at: https://www.mdpi.com/article/10.3390/pharmaceutics14010080/s1, Figure S1: In vitro cell viability detected by MTT and NRU assays after 72 h coincubation of PLGA-77KL-NPs (blank NPs without the organoselenium compound) with DOX in MCF-7 and NCI/ADR-Res cell lines. Data are expressed as the mean of three independent experiments ± SE. Figure S2: Combination index values for the association of PLGA-77KL-NPs (blank NPs without the organoselenium compound) with DOX in MCF-7 and NCI/ADR-Res cell lines.
