# Supplementary material for: Overcoming MDR by Associating Doxorubicin and pH-Sensitive PLGA Nanoparticles Containing a Novel Organoselenium Compound—An In Vitro Study
Source: Pharmaceutics. 2021 Dec 29;14(1):80. doi: 10.3390/pharmaceutics14010080 (PMC8779681; doi:10.3390/pharmaceutics14010080)
Supplement: Supplementary file 1 [file pharmaceutics-14-00080-s001.zip › pharmaceutics-1491005-supplementary.pdf]

pharmaceutics

Supplementary Materials

Overcoming MDR by Associating Doxorubicin and pH-Sensitive PLGA Nanoparticles Containing a Novel Organoselenium Compound—An *In Vitro* Study

Letícia Bueno Macedo [1,2], Daniele Rubert Nogueira-Librelotto [1,2,*], Daniela Mathes [1,2], Josiele Melo de Vargas [2], Raquel Mello da Rosa [3], Oscar Endrigo Dorneles Rodrigues [3], Maria Pilar Vinardell [4,5], Montserrat Mitjans [4,5*] and Clarice Madalena Bueno Rolim [1,2]

1 Programa de Pós-Graduação em Ciências Farmacêuticas, Universidade Federal de Santa Maria, Av. Roraima 1000, Santa Maria 97105-900, RS, Brazil; leticiabuenomacedo@gmail.com (L.B.M.); danielamathes1609@gmail.com (D.M.); clarice.rolim@gmail.com (C.M.B.R.)
2 Departamento de Farmácia Industrial, Universidade Federal de Santa Maria, Av. Roraima 1000, Santa Maria 97105-900, RS, Brazil; josydivargas@gmail.com
3 Departamento de Química, Universidade Federal de Santa Maria, Av. Roraima 1000, Santa Maria 97105-900, RS, Brazil; raquelmello.rosa@gmail.com (R.M.d.R.); rodriguesoed@gmail.com (O.E.D.R.)
4 Departament de Bioquimica i Fisiologia, Facultat de Farmacia i Ciències de l'Alimentaciò, Universitat de Barcelona, Av. Joan XXIII 27-31, 08028 Barcelona, Spain; mpvinardellmh@ub.edu
5 Institute of Nanocience and Nanotechnology (IN2UB), Universitat de Barcelona, Av. Diagonal 465, 08028 Barcelona, Spain
* Correspondence: librelotto.daniele@ufsm.br (D.R.N.-L.); montsemitjans@ub.edu (M.M.)

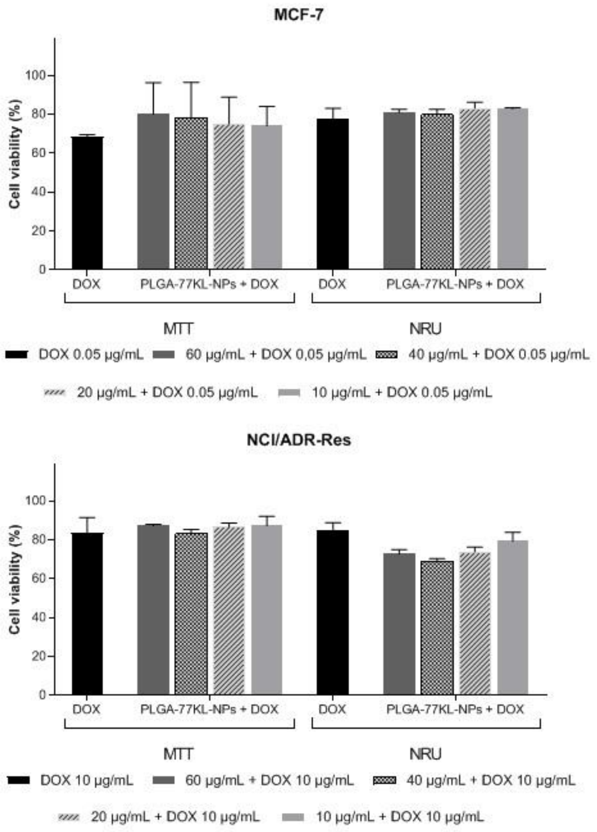

Figure S1. In vitro cell viability detected by MTT and NRU assays after 72 h coincubation of PLGA-77KL-NPs (blank NPs without the organoselenium compound) with DOX in MCF-7 and NCI/ADR-Res cell lines. Data are expressed as the mean of three independent experiments ± SE.

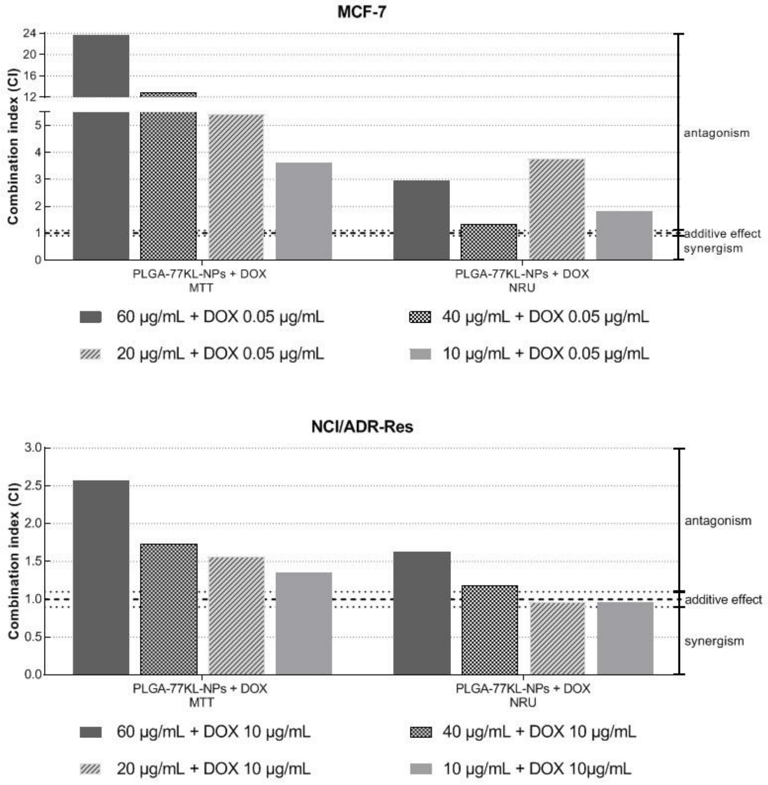

Figure S2. Combination index values for the association of PLGA-77KL-NPs (blank NPs without the organoselenium compound) with DOX in MCF-7 and NCI/ADR-Res cell lines.
